# How much do plastic surgeons add to the closure of myelomeningoceles?

**DOI:** 10.1007/s00381-017-3674-9

**Published:** 2017-12-08

**Authors:** Rhian Bevan, Nicholas Wilson-Jones, Imran Bhatti, Chirag Patel, Paul Leach

**Affiliations:** 1 University Hospital of Wales College of Medicine, Cardiff, UK; 2 Welsh Centre for Burns and Plastic Surgery, Swansea, UK; 3 Department of Paediatric Neurosurgery, University Hospital of Wales, Cardiff, UK

**Keywords:** Myelomeningocele repair, Spinal dysraphism, Neural tube defect, Plastics

## Abstract

**Purpose:**

This study reviews the outcomes of children undergoing myelomeningocele (MMC) repair in the paediatric neurosurgical department in Cardiff. These procedures are historically performed by paediatric neurosurgeons with occasional support from plastic surgeons for the larger lesions. We reviewed the postoperative outcomes over a 9-year period to assess the efficacy of having a plastic surgeon present at all MMC closures.

**Methods:**

Analysis of a prospectively collected database of all MMC closures performed at University Hospital Wales from April 2009 to August 2017 was used. Comparison was made with the published literature especially with regard to complications.

**Results:**

Thirty-one children, 13 males and 18 females, underwent MMC closure over the 9-year period. Twenty-four (77.4%) defects were closed by direct approximation. Seven patients (22.5%) required a more complex plastic procedure to obtain closure. Two patients (6.5%) had a wound complication, one wound infection and one flap edge necrosis both healing with dressings alone. Two patients had cerebrospinal fluid (CSF) leaks that responded to ventriculo-peritoneal shunting. Two patients died from unrelated conditions during the study period.

**Conclusion:**

In our series, 7/31 (22.5%) cases involved a more complex closure in keeping with the literature. The authors feel that having the plastic surgeon at all closures has led to a low wound complication rate.

## Introduction

Myelomeningocele (MMC) is the commonest congenital malformation of the central nervous system compatible with life [1]. It occurs due to the unsuccessful closure of the neural tube, most commonly affecting the lumbo-sacral region. Increased uptake of pre-conceptual folic acid has reduced the incidence of neural tube defects (NTD) in the UK [2], although in Wales, there has been an increase in live births over the last decade [3]. Following the diagnosis of MMC on prenatal ultrasound, the foetal management team request specialist neurosurgical counselling. For the mothers continuing with pregnancy, they are electively admitted for induction of labour 1 week prior to their due date. Repair is generally performed within 72 h of birth. Previous management of MMC repair has traditionally been solely neurosurgical, with additional plastic surgical involvement being reserved for larger lesions. In Cardiff, these closures are performed jointly with a paediatric neurosurgeon and paediatric plastic surgeon. We reviewed the outcomes of this cohort of patients over the last 9 years since joint operating became common place.

## Methods

All 31 patients that underwent MMC repair at University Hospital of Wales between April 2009 and August 2017 were inputted into a prospective database during the patient’s hospital stay. Age at closure, the level of lesion and complications were documented prospectively.

We looked at the various methods of closure for the cohort of patients and how often a more complex flap-based closure was needed. We also compared the outcomes, especially with regard to complications, to the current published literature to assess the efficiency of involving plastic surgeons in MMC closures.

## Results

Thirty-one children were operated on between April 2009 and August 2017 (Table 1). Of the 30, 13 (42%) were males and 18 (58%) females. Nighty percent underwent closure within 72 h of birth, with 63% operated on within the first 48 h. Seven (22.5%) repairs required a flap procedure to obtain closure with the remaining 24 (77.4%) defects closed by direct skin approximation. One patient underwent bilateral latissimus dorsi flaps with perforator-based V to Y skin closure, one patient had bilateral rhomboid skin flaps and five patients required a unilateral rhomboid flap to obtain closure. A ventriculo-peritoneal shunt (VP) was required in 21 cases (68%).

**Table 1:** Primary dataset. Myelomeningocele repairs (April 2009–March 2017)

Patient ID	Age at repair (days)	Site of MMC	Plastics procedure	Postoperative complications	VP shunt required
1	1	Lumbar	–	–	Y
2	2	Lumbar	Y	–	Y
3	1	Thoraco-lumbar	–	CSF leak	Y
4	3	Lumbar	–	–	Y
5	3	Lumbo-sacral	–	–	Y
6	3	Lumbar	–	–	N
7	3	Thoraco-lumbar	Y	–	Y
8	5	Lumbo-sacral	–	–	Y
9	1	Thoraco-lumbar	–	–	N
10	2	Lumbo-sacral	–	–	N
11	1	Lumbo-sacral	–	–	Y
12	1	Lumbo-sacral	–	–	N
13	2	Lumbar	–	–	N
14	1	Thoraco-lumbar	Y	Flap necrosis	Y
15	1	Lumbo-sacral	Y	CSF leak	Y
16	3	Lumbo-sacral	–	–	Y
17	2	Lumbo-sacral	–	–	N
18	3	Lumbo-sacral	–	Wound infection	N
19	2	Thoraco-lumbar	–	–	Y
20	3	Lumbo-sacral	Y	–	Y
21	1	Lumbo-sacral	–	–	N
22	4	Sacral	–	–	N
23	1	Lumbo-sacral	–	–	Y
24	1	Lumbo-sacral	Y	–	Y
25	2	Lumbo-sacral	Y	–	Y
26	4	Sacral	–	–	Y
27	3	Lumbo-sacral	–	–	Y
28	2	Lumbo-sacral	–	–	Y
29	2	Lumbo-sacral	–	–	Y
30	1	Lumbo-sacral	–	–	Y
31	1	Lumbo-sacral	–	–	N

One patient developed a wound infection which was treated successfully with appropriate antibiotics. The patient that underwent a double rhomboid flap closure developed partial wound breakdown at the suture edge where the two flaps met due to distal flap tip necrosis. However, this was successfully managed conservatively with dressings. Two patients developed a cerebrospinal fluid leak day 1 post closure, both responded immediately to VP shunt placement.

There have been two deaths due to unrelated conditions during the series. One patient had respiratory failure secondary to sepsis resulting in multi-organ failure at 11 days old. The second patient was palliated and died at 2 years and 6 months from respiratory sepsis.

## Discussion

Tension-free closure of the skin must be achieved to provide soft tissue protection for the neural elements and dural closure. This will help prevent wound breakdown, CSF leak and wound infection. Techniques used to achieve primary closure range from simple undermining of the skin with primary approximation to complex plastic surgical procedures. Historically, up to 75% [4–7] of defects are closed primarily and do not require additional plastic surgical input. Simple skin approximation requires less operative time and results in a significant reduction in blood loss. In our primary dataset, 77.4% of defects were closed by direct skin approximation.

Previous literature has suggested that the diameter of the lesion should dictate its method of closure; traditionally flap reconstruction is recommended for defects that are > 5 cm in diameter [7, 8]. Increasingly, a more tailored approach incorporating the location, shape and area, in addition to lesion length, has been reported [5, 9, 10]. Decisions on closure made on an individual basis instead of the width of the defect yield lower complication rates [5]. The condition of the skin surrounding the lesion is also an important variable to consider when deciding upon the method of closure.

Plastic surgeons contribute experience and knowledge in tissue handling. They perform direct skin closure after perforator preserving dissection with loupe magnification that conserves vascular supply to the wound edges. We believe that this improves the on-table decision-making regarding which defects can be closed by direct approximation or reverting to a flap for closure.

The perforator preserving dissection in combination with an increased understanding of the ‘junctional zone’, the thickened area of dense subcutaneous tissue at the junction of the arachnoid, dura and dermis [11], has allowed higher-tension closure in the deeper tissues without de-vascularisation of the skin edge.

Complications of MMC closure in the first 30 days postoperatively are most commonly wound complications such as dehiscence and infection [4, 12]. The literature reports complication rates between 7.7 and 33% [13–18] for this vulnerable group of patients. Our overall complication rate was 12.9%. However, only two of these were as a direct result of wound complications, one with flap edge necrosis and one due to infection. The two CSF leaks were primarily driven by the development of hydrocephalus and resolved immediately after the insertion of a VP shunt. This is certain in keeping with the published literature for MMC closures.

## Conclusion

The increased expertise of a plastic surgeon being involved with all MMC closures allows a tailored approach with on-table multidisciplinary decision-making. We believe this leads to as robust, durable aesthetically improved wound closure with very low wound complication rates.
